# Influence of the Serotonin Transporter 5HTTLPR Polymorphism on Symptom Severity in Irritable Bowel Syndrome

**DOI:** 10.1371/journal.pone.0054831

**Published:** 2013-02-05

**Authors:** Rocchina Colucci, Dario Gambaccini, Narcisa Ghisu, Giuseppe Rossi, Francesco Costa, Marco Tuccori, Nicola De Bortoli, Matteo Fornai, Luca Antonioli, Angelo Ricchiuti, Maria Gloria Mumolo, Santino Marchi, Corrado Blandizzi, Massimo Bellini

**Affiliations:** 1 Division of Pharmacology and Chemotherapy, Department of Clinical and Experimental Medicine, University of Pisa, Pisa, Italy; 2 Unit of Gastroenterology, Department of Translational Medicine, University of Pisa, Pisa, Italy; 3 Unit of Epidemiology and Biostatistics, Institute of Clinical Physiology, National Research Council, Pisa, Italy; University of California, Los Angeles, United States of America

## Abstract

5HTTLPR polymorphism of serotonin transporter yields short (S) and long (L) alleles. SS and LS genotypes are associated with reduced expression of serotonin transporter. This cross-sectional study investigated the association of 5HTTLPR with symptom severity of irritable bowel syndrome (IBS). Patients with IBS (Rome III) and healthy controls were included. Genomic DNA was extracted from saliva, and 5HTTLPR alleles were assessed by polymerase chain reaction. IBS symptom severity was evaluated by means of IBS-SSS questionnaire. Two hundreds and four IBS patients (159 females; mean age: 39.6±12.3 years; 106 with constipation: C-IBS; 98 with diarrhea: D-IBS) and 200 healthy controls (154 females; mean age: 40.4±15.8 years) were enrolled. The overall IBS-SSS value was higher in LS/SS than LL patients (319.0±71.5 versus 283.8±62.3; P = 0.0006). LS/SS patients had also higher values of abdominal pain (59.7±21.0 versus 51.0±18.8; P = 0.020) and bowel dissatisfaction (80.1±23.9 versus 70.5±22.8; P = 0.035). The overall IBS-SSS values in C-IBS and D-IBS patients were 317.2±68.3 and 296.1±71.4, respectively (P = 0.192), with significantly higher values for abdominal distension (65.0±24.4 versus 51.4±24.8; P = 0.0006), but not for bowel dissatisfaction (80.5±21.7 versus 72.9±25.7; P = 0.138). Frequencies of 5HTTLPR genotypes did not differ significantly when comparing IBS patients (overall or upon stratification in C-IBS and D-IBS) with healthy controls. In conclusion, the LS and SS genotypes are significantly correlated with IBS symptom severity, although their possible direct causal role remains to be proven. In addition, the present findings do not support an association of 5HTTLPR with IBS or its clinical presentation in terms of bowel habit predominance.

## Introduction

Irritable bowel syndrome (IBS) is a functional digestive disorder characterized by abdominal pain/discomfort and changes in bowel habit (constipation and/or diarrhoea), which can be associated with alterations of gastrointestinal (GI) transit, visceral hypersensitivity and psychopathological comorbidities. [1], [2] Owing to its chronicity and substantial lack of effective cures, IBS results in a significant social burden, with a loss of work productivity, impaired quality of life, and a remarkable use of health care resources. [3].

From a pathogenic standpoint, IBS is a complex, heterogeneous disorder, whose development is widely considered as multifactorial in nature. [4], [5] Based on current evidence, a gene-environment paradigm has been proposed for IBS, whereby a combination of genetic determinants and environmental factors gives rise to alterations in GI sensation and motor functions, that ultimately account for the occurrence of symptoms. [6], [7] In this context, studies on families or twins have given support to the hypothesis of a genetic background in IBS and, consequently, intensive efforts are being made to evaluate whether specific polymorphisms of a number of candidate genes are pathogenically associated with IBS. [6], [8] Furthermore, since genetic factors do not act necessarily as risk factors, but they can also impact on clinical phenotypes, great attention is being paid also to possible relationships linking genetic polymorphisms to intermediate phenotypes (e.g., GI transit and pain threshold to rectal distension) or clinical presentation/severity. [9], [10], [11].

The involvement of serotonin (5-HT) in the pathophysiology of IBS is also an area of active investigation, owing to its presence in both GI tract and brain. Indeed, 5-HT acts as a main regulator of GI motility, secretion and sensory signalling, and plays also a pivotal role in the control of mood at level of the central nervous system (CNS). [12], [13] The magnitude and duration of 5-HT actions depend mostly on 5-HT transporter (SERT), which mediates the extracellular reuptake of 5-HT, thus ensuring its recycling and catabolic breakdown. [14], [15] Since abnormalities in 5-HT reuptake have the potential of altering the enteric serotonergic signalling, leading to motor, secretory and sensory gut dysfunctions, it has been suggested that genetic or epigenetic SERT variations might contribute to the pathogenesis or clinical presentation of IBS. [7], [16].

The promoter region of human SERT gene contains a polymorphism, designated as ‘5-HT transporter length polymorphic region’ (5HTTLPR), which consists of a 44-base pair deletion/insertion, resulting in a short (S) and long (L) allele. [17], [18] The implications of this polymorphism in IBS are currently under evaluation since, as compared with LL genotype, SS or SL genotypes are associated with lower levels of SERT mRNA transcripts, and thereby lower levels of SERT expression and lower efficiency of 5-HT reuptake. [19], [20], [21] A meta-analysis of association studies has not supported a pathogenic link of 5HTTLPR with IBS. [22] By contrast, some studies have found a significant association when IBS patients were stratified by their predominant bowel habit, [23] or were investigated for pain sensation at rectal distension or somatic symptom scores. [10], [24] However, whether 5HTTLPR exerts any influence on symptom severity in IBS remains undetermined. Accordingly, the present study was carried out with the primary purpose of investigating a possible association of 5HTTLPR polymorphism with overall symptom severity in IBS patients.

## Methods

### Study Aims

The primary objective was to examine the association of 5HTTLPR with the overall symptom severity in IBS patients. The secondary objectives were: 1) to evaluate the association of 5HTTLPR with the severity of different symptoms; 2) to evaluate the overall symptom severity and the severity of different IBS symptoms in patients with predominance of constipation (C-IBS) or diarrhoea (D-IBS); 3) to evaluate the association of 5HTTLPR with IBS in all IBS patients, as well as in the subgroups of C-IBS or D-IBS, by comparisons with healthy controls.

### Study Design and Population

This study was conducted on native Italians of Caucasian origin, gender of either sex, age equal or higher than 18 years, and diagnosis of IBS according to Rome III criteria, [1] referred consecutively to the Unit of Gastroenterology from June 2009 to December 2011. Exclusion criteria included: severe organic diseases; history of major abdominal surgery; severe psychiatric disorders; significant alterations of blood chemistry, haematology, and urine analysis; organic lesions at colonoscopy, barium enema, or abdominal ultrasonography; lactose intolerance; treatment with any drug, employed for IBS or affecting the digestive system or CNS, within the previous month. IBS patients with alternation of diarrhea and constipation were also excluded, since changes in the clinical phenotype are common in this subpopulation. [25] To evaluate the association of 5HTTLPR with IBS, healthy volunteers, consisting of native Italians of Caucasian origin, gender of either sex, and age equal or higher than 18 years, were also enrolled. The exclusion criteria included: body mass index lower than 19 or higher than 25 kg/m^2^; unremarkable medical history; any functional or organic disease; history of major abdominal surgery; severe psychiatric disorders; food intolerance; any hematologic abnormality.

### Experimental Design

At least two samples of saliva were collected from each IBS patient and healthy volunteer in different days and stored at −20°C. All subsequent analytical procedures were performed at the Division of Pharmacology and Chemotherapy. The severity of IBS symptoms was evaluated by the questionnaire IBS-Symptom Severity Score (IBS-SSS). [26] Then all patients received the standard of care. The study protocol was approved by the Ethical Committee of Pisa and was carried out in accordance with the Helsinki Declaration (Edinburgh revision, 2000). A signed informed consent was obtained from each participant.

### 5HTTLPR Polymorphism Assay

Genomic DNA was isolated from saliva by the alkaline method using QIAamp DNA blood MINI kit (Qiagen, Milano, Italy), quantified by spectrophotometry, and visualized by agarose gel electrophoresis. At the first run, success rates of 97.5% and 95.5% were obtained for DNA extraction in IBS patients and healthy controls, respectively. Additional samples of saliva were then employed or collected again from subjects with extraction failure, and success rates of 100% were achieved at the second run of genomic DNA extraction. In order to avoid and properly manage genotyping errors, for each batch the following cautions were taken as suggested by Pompanon et al. [27]: 1) the laboratory staff was blinded to sample allocation; 2) blanks and duplicate samples of control DNA were included; 3) each sample was assayed in duplicate; 4) cases of genotyping failure in at least one duplicate were resolved by assaying DNA extracted from additional samples of saliva; 5) care was taken to verify whether the genotype distribution was in Hardy-Weinberg equilibrium. The 5HTTLPR polymorphism was typed by polymerase chain reaction (PCR) using the following oligonucleotide primers: forward (LPR-F), 5′-GGCGTTGCCGCTCTGAATGC-3′, spanning from nucleotide –1416 to –1397; reverse (LPR-R), 5′-GAGGGACTGAGCTGGACAACCAC-3′, spanning from –910 to –888. PCR was performed with the GC-Rich PCR system (Roche Molecular Biochemicals), in a 50-µl reaction containing 1 µl of genomic DNA (20–50 ng), 25 µl of DNAase free H_2_O, 1 µl of 10 mM dNTP, 1 µl of 10 mM LPR-F primer, 1 µl of 10 mM LPR-R primer, 10 µl of BUFFER GC resolution, 10 µl of BUFFER 5× DMSO and 1 µl of AmpliTaq polymerase enzyme (ROCHE Diagnostics, Milano, Italy). DNA was denatured at 94°C for 3 min and subjected to 35 cycles of 1 min of denaturation at 94°C, 1 min of annealing at 55°C, 1 min of extension at 72°C, and 7 min of final extension at 72°C. Amplification products were resolved by electrophoresis on 2% agarose gel, and visualized with ethidium bromide staining. The image was acquired by means of a Kodak Image Station 440. The alleles containing the S or L variant of 5HTTLPR yielded PCR products of 528 and 572 base pairs, respectively. Based on PCR results, the enrolled subjects were classified as having the LL, LS or SS genotype.

### Evaluation of Symptom Severity

The IBS-SSS questionnaire takes into account the following items: a) presence and severity of abdominal pain or discomfort; b) frequency of abdominal pain or discomfort; c) presence and severity of abdominal distension; d) degree of satisfaction of defecatory behaviour; e) degree of interference of IBS symptoms with daily lifestyle. Each of the above items generates a maximum score of 100, leading to an overall score of 500. [26].

### Endpoints

Primary endpoint: overall IBS-SSS value, to compare LL versus LS/SS genotypes in patients. Secondary endpoints: 1) value of each IBS-SSS item, to compare LL versus LS/SS genotypes in patients; 2) overall IBS-SSS value, to compare C-IBS versus D-IBS patients; 3) value of each IBS-SSS item, to compare C-IBS versus D-IBS patients; 4) frequencies of LL and LS/SS genotypes, to compare IBS patients with healthy volunteers; 5) frequencies of LL and LS/SS genotypes, to compare C-IBS patients, D-IBS patients and healthy volunteers.

### Statistical Analysis

Data were expressed as mean ± standard deviation (SD) for continuous variables, and as percentage for qualitative variables. Comparisons between groups were performed by analysis of variance (ANOVA) and the non-parametric Wilcoxon unpaired test for continuous variables, and by the Pearson’s chi-square or the Fisher’s exact test for categorical variables. Adjustments for multiple comparisons were carried out by the Bonferroni correction. Comparisons between groups for IBS-SSS values were also performed by a two-factor (genotype and IBS subgroups) ANOVA, and the two-factor interaction was evaluated. The patients sample size was determined to evaluate a between-groups (LL versus LS/SS genotype) difference of 30 in the mean overall IBS-SSS value, with a standard deviation of 65, a first type error of 0.05 and a power of 0.80. A 2-sided P value <0.05 was considered to be statistically significant. Statistical analysis was performed by JMP 4.0 (SAS Institute Inc., Cary, NC, U.S.A.) and StatXact-4 (Cytel Software Corporation, Cambridge, MA, U.S.A.).

## Results

### Influence of 5HTTLPR Genotypes on IBS Symptom Severity

Two hundreds and four consecutive patients (45 males, 159 females; mean age: 39.6±12.3 years), with a diagnosis of C-IBS (6 males, 100 females; mean age: 41.2±12.9 years) or D-IBS (39 males, 59 females; mean age: 38.0±11.5 years), were enrolled to assess the relationship between 5HTTLPR genotypes and IBS symptom severity. The C-IBS and D-IBS subgroups differed significantly for male/female proportion (P<0.0001), but not for age. For the purpose of analyzing the primary and secondary endpoints, the subjects with SS and LS genotypes were pooled (LS/SS), since the S allele has been reported to be dominant and both SS and LS genotypes have been associated with a lower SERT expression and reduced efficiency of 5-HT uptake, as compared with the LL genotype. [17], [18] However, in order to perform additional exploratory evaluations, subjects with genotypes LS and SS were also analysed as separate groups.

The overall mean symptom severity, as assessed by IBS-SSS, was significantly higher in patients with LS/SS than LL genotype (Table 1). When comparing the mean values obtained for each IBS-SSS item, ‘abdominal pain severity’ and ‘bowel dissatisfaction’ were significantly higher in patients with LS/SS than LL genotype, while no significant differences were found with regard for the ‘occurrence of pain over the preceding 10 days’, ‘abdominal distension severity’, and ‘interference with quality of life’ (Table 1). These findings were confirmed also when the P values were adjusted for multiple comparisons by the Bonferroni correction. Of note, an exploratory analysis showed that, when comparing patients with LS and SS genotypes as separate groups, there were no significant differences with regard for the overall symptom severity (P = 0.496) and single-item IBS-SSS values (abdominal pain severity, P = 0.935; occurrence of pain over the preceding ten days, P = 0.348; abdominal distension severity, P = 0.968; bowel dissatisfaction, P = 0.309; interference with quality of life, P = 0.851).

**Table 1:** Mean values (± standard deviation) of overall and single-item IBS-SSS in patients with LL versus LS/SS genotype.

	LL (n = 69)	LS/SS (n = 135)	P	P (*)
Abdominal pain severity	51.0±18.8	59.7±21.0	0.004	0.020
Occurrence of pain over the preceding 10 days	50.4±26.4	55.4±29.8	0.089	0.445
Abdominal distension severity	56.4±24.7	59.5±25.9	0.401	1,000
Bowel dissatisfaction	70.5±22.8	80.1±23.9	0.007	0.035
Interference with quality of life	55.7±25.9	62.1±28.3	0.116	0.580
**Overall score**	283.8±62.3	319.0±71.5	0.0006	

(*) P values after Bonferroni correction.

Symptom severity was assessed also in C-IBS and D-IBS patients. The overall mean IBS-SSS values were found to be significantly higher in the C-IBS than D-IBS subgroup, irrespectively of their 5HTTLPR genotype (Table 2). Likewise, when evaluating symptom severity by single items, the score values for ‘abdominal distension severity’ and ‘bowel dissatisfaction’ were significantly higher in C-IBS than D-IBS patients (Table 2). However, upon application of Bonferroni correction, the significance of differences in terms of overall symptom severity and bowel dissatisfaction were lost (P = 0.192 and 0.138, respectively), while the significance of differences concerning abdominal distension severity was confirmed (P = 0.0006).

**Table 2:** Mean values (± standard deviation) of overall and single-item IBS-SSS in IBS patients with predominance of constipation (C-IBS) or diarrhea (D-IBS).

	C-IBS (n = 106)	D-IBS (n = 98)	P	P (*)
Abdominal pain severity	57.5±20.8	55.9±20.6	0.563	1,000
Occurrence of pain over the preceding 10 days	56.6±30.5	53.3±27.9	0.424	1,000
Abdominal distension severity	65.0±24.4	51.4±24.8	0.0001	0.0006
Bowel dissatisfaction	80.5±21.7	72.9±25.7	0.023	0.138
Interference with quality of life	57.6±27.6	62.5±27.5	0.210	1.000
Overall score	317.2±68.3	296.1±71.4	0.032	0.192

(*) P values after Bonferroni correction.

There was a lack of significant interaction between bowel habits (i.e., C-IBS and D-IBS) and 5HTTLPR genotypes, indicating no effect modification and independent effects of bowel habits and genotypes on symptom severity (interaction P values: 0.297 for overall IBS-SSS value; 0.078 for abdominal pain severity; 0.481 for occurrence of pain over the preceding 10 days; 0.811 for abdominal distension severity; 0.379 for bowel dissatisfaction; 0.406 for interference with quality of life).

### Association of 5HTTLPR Polymorphism with IBS

In order to evaluate whether 5HTTLPR polymorphism is associated with IBS in the present group of patients, a comparison was made with a group of healthy controls. For this purpose, 200 healthy volunteers (46 males, 154 females; mean age: 40.4±15.8 years) were enrolled in the study. They did not differ significantly in terms of male/female proportion and mean age from the group of IBS patients.

The frequencies of 5HTTLPR genotypes in IBS patients and healthy volunteers are displayed in Table 3. Care was taken to verify that the genotype distribution in healthy volunteers was in Hardy-Weinberg equilibrium (chi-square = 0.21, P = 0.646). In order to allow comparisons with previous observations, the LS and SS genotypes were analyzed either separately or as a pooled group (LS/SS). In both cases, the frequencies of LL, LS, SS or LS/SS genotypes in IBS patients did not differ significantly from those estimated in healthy volunteers (Table 3). Likewise, genotypes frequencies did not differ significantly when the comparisons were conducted on IBS patients after their stratification into two subgroups, according to the predominance of constipation or diarrhea (Table 4).

**Table 3:** Frequencies of 5HTTLPR genotypes in IBS patients and healthy volunteers (HV).

	IBS (n = 204)	HV (n = 200)	P
**LL**	69 (33.8%)	60 (30.0%)	0.657 (a)
**LS**	101 (49.5%)	102 (51.0%)	
**SS**	34 (16.7%)	38 (19.0%)	
**LS/SS**	135 (66.2%)	140 (70.0%)	0.455 (b)

(a) comparison by LL, LS and SS subgroups.

(b) comparison by LL and LS/SS subgroups.

**Table 4:** Frequencies of 5HTTLPR genotypes in IBS patients with predominance of constipation (C-IBS) or diarrhea (D-IBS) and healthy volunteers (HV).

	C-IBS (n = 106)	D-IBS (n = 98)	HV (n = 200)	P
**LL**	36 (33.9%)	33 (33.7%)	60 (30.0%)	0.594 (a)
**LS**	56 (52.8%)	45 (45.9%)	102 (51.0%)	
**SS**	14 (13.3%)	20 (20.4%)	38 (19.0%)	
**LS/SS**	70 (66.1%)	65 (66.3%)	140 (70.0%)	0.711 (b)

(a) comparison by LL, LS and SS subgroups.

(a) comparison by LL and LS/SS subgroups.

## Discussion

Great attention is currently revolving around the pathogenic bases and pathophysiological mechanisms accounting for the clinical manifestations of IBS, [4], [5] and there is consistent evidence suggesting an influence of genetic factors on this disorder. [6], [8] In this context, our study provides the first evidence that 5HTTLPR impacts on the severity of IBS symptoms. In particular, our findings point out a higher degree of symptom severity in patients with at least one S allele, and suggest that the S variant affects mainly the severity of abdominal pain and bowel dissatisfaction. Of note, our exploratory analysis showed that IBS-SSS values did not differ significantly when comparing subgroups with LS and SS genotypes, thus suggesting that the group of patients with pooled LS/SS genotypes was a homogeneous population.

The contention that the LS/SS genotypes can increase the severity of IBS symptoms, with particular regard for abdominal pain, is supported by previous investigations. In particular our findings are in line with data from Camilleri et al. [10] who found that the LS/SS genotypes are associated with increased pain sensation at rectal distension. Taken together, these observations [10] and our results are consistent with the notion that the S allele is expected to impair the efficiency of 5-HT reuptake with a consequent prolonged and enhanced activation of serotonergic pathways mediating abdominal pain sensation. Therefore, it can be argued that the reduced 5-HT re-uptake could induce an overstimulation of extrinsic nerve afferents resulting in neuronal sensory sensitisation, which might occur at peripheral, spinal, or even higher CNS levels. [23], [28] In this regard, an interesting study by Fukudo et al. [29], aimed at evaluating the link between 5HTTLPR and differential activation of brain regions by colorectal distention in humans, displayed that individuals with a weak function of serotonin transporter (i.e. S/S genotype) responded to signals from gut with higher intensity in emotion-regulating brain regions, thus suggesting that this functional gene polymorphism may partly predict the individual effects of selective serotonin reuptake inhibitors on visceral pain. [29].

Whether the influence of 5HTTLPR on abdominal pain results mainly from peripheral or central mechanisms, or both, is presently unknown and remains open to future investigations. However, some evidence suggests that central mechanisms may contribute to the enhanced pain perception in IBS patients with LS/SS genotypes: 1) specific brain regions of IBS patients and healthy controls are subjected to differential activation upon application of rectal stimuli; [30] 2) brain regions highly related to pain recognition and emotion (e.g. anterior cingulated cortex) were found to be more activated by distension of descending colon in IBS patients, [9] and altered interactions between anterior cingulated cortex and amygdala have been reported in subjects with LS/SS genotypes; [31] 3) individuals with the SS genotype were more likely to activate these CNS regions, thus suggesting that the S variant can be relevant in brain activation after colorectal distension in IBS patients. [11] Further support to the contribution of central nervous mechanisms on the association of 5HTTLPR with symptom severity in IBS comes from the observations that: 1) in IBS patients, the LS/SS genotypes were associated with high values of somatic symptom scores, suggesting that 5-HTTLPR is implicated in the responses of IBS patients to stress; [24] 2) individuals with the S allele display higher vulnerability to stressful life events and are more prone to develop depressive symptoms. [32]. In this respect, to assess whether the influence of S allele on abdominal pain severity, could be related to differences in patients’ psychopathological traits would be of high interest, and this issue deserves future investigations.

In the present study, when the P values were adjusted for multiple comparisons, the analysis for secondary endpoints revealed that the overall symptom severity did not differ in C- and D-IBS patients, while the analysis of single severity items displayed significant differences only for the severity of abdominal distension. Moreover, our analysis showed a lack of significant interaction between bowel habits and 5HTTLPR genotypes. These findings were not completely unexpected, since C-IBS patients are known to complain of abdominal distension more than D-IBS patients. [33] In addition, and most importantly, these data argue against the possibility that the differential influences of 5HTTLPR genotypes on abdominal pain severity might result from differences in bowel habit.

The influence of 5HTTLPR on IBS symptom severity and, particularly, on abdominal pain severity, might have interesting implications for the effectiveness and/or safety of drugs proposed for the therapeutic management of this syndrome. SERT is a specific target of ‘selective serotonin reuptake inhibitors’ (SSRIs) and there is evidence linking 5HTTLPR to the efficacy and safety of these drugs in patients with depression. [34], [35] It is being also appreciated that SSRIs can exert beneficial effects on IBS, with particular regard for the control of abdominal discomfort or pain, [16], [36] but whether SERT polymorphisms may influence such therapeutic responses remains unknown.

To date, most of the studies on the impact of 5-HT genetics on IBS have investigated the associations of 5HTTLPR with its pathogenesis or clinical presentation. In this regard, the present study was conducted also with the secondary objective of evaluating whether any of such associations could be highlighted in our IBS patients. For this purpose, the frequencies of their 5HTTLPR genotypes were compared with those of healthy controls, selected with similar demographic characteristics, and no significant association was found either for the overall IBS group or for the subgroups of C-IBS and D-IBS patients. Our results are consistent with the majority of previous studies [6], [22] which failed in finding a link between 5HTTLPR and IBS. Moreover, while most authors did not find specific links of 5HTTLPR with C-IBS or D-IBS, [24], [37], [38], [39], [40] others have reported significant associations of SS with D-IBS, [23], [41] SS with C-IBS, [42] or LL with C-IBS. [10], [21], [43] Among the variety of factors which might account for the heterogeneity of these observations, the most relevant relies perhaps in the different distribution of 5HTTLPR genotypes among different populations. In particular, the allele frequencies between Caucasian and Asian populations are different, since S allele is found in 42% of Caucasians and in 79% of Asians. [44] Furthermore, significant variations in the frequency of 5HTTLPR genotypes can be found even in populations sharing the same Caucasian origin, but belonging to distinct ethnicities. [44] In this respect, both the IBS patients and healthy volunteers evaluated in our study were highly homogeneous, as we enrolled only white Caucasian subjects of Italian origin.

In conclusion, the present study suggests that the LS and SS genotypes are significantly correlated with IBS symptom severity, although their possible direct causal role remains to be proven. In addition, the present findings do not support an association of 5HTTLPR with IBS or its clinical presentation in terms of bowel habit predominance.
